# Grape Skin Polysaccharides Alleviate Type 2 Diabetic Rats via Gut Microbiota and Nontargeted Metabolism Alterations

**DOI:** 10.3390/foods14234132

**Published:** 2025-12-02

**Authors:** Wei Li, Xinyao Han, Wei Wang, Shihao Huang, Mingxun Ai, Tongle Sun, Haoran Jiang, Hongji Zeng, Yuhang Li

**Affiliations:** College of Food Science and Pharmacy, Xinjiang Agricultural University, Urumqi 830052, China; liweili2025@163.com (W.L.);

**Keywords:** grape skin polysaccharides, T2D, hypoglycemic, dysbiotic intestinal flora, untargeted metabolomics

## Abstract

This study investigates the therapeutic potential of grape skin polysaccharides (GSP) for type 2 diabetes (T2D). Employing a T2D model developed via a high-fat diet combined with STZ, three intervention groups were established: low-dose GSP (25 mg/kg), high-dose GSP (100 mg/kg), and metformin control (300 mg/kg). Following a 30-day oral administration period, marked enhancements in body weight and glucose/lipid metabolic parameters were noted in both the high-dose GSP group and the metformin-treated cohort. Specifically, compared with the model group, high-dose GSP improved insulin resistance by 48.48%, increased hepatic glycogen content by 63.38% and HDL–C levels by 13.16%, while reducing TG, TC, and LDL–C by 65.5%, 20.80%, and 32.63%, respectively. GSP also enhanced GSH–Px activity by 10.15% and SOD activity by 26.48%, while reducing MDA levels by 30.91%, thereby alleviating pathological damage in the liver, kidneys, and intestines. These results suggested that the regulatory effect of GSP is concentration-dependent. GSP also regulated gut microbiota by not only reducing Thermodesulfobacteriota and increasing Bacillota/Bacteroidetes abundance, but also enhancing acid-producing bacteria to elevate short-chain fatty acid (SCFA) levels, thereby further improving insulin sensitivity. Collectively, these preclinical data support the potential of GSP as a functional food ingredient or adjunct therapy for T2D management, pending further clinical validation.

## 1. Introduction

As a chronic metabolic disease with significant epidemiological implications, diabetes mellitus has emerged as the third leading contributor to global disease burden among non-communicable disorders [1,2]. Notably, type 2 diabetes (T2D) mellitus accounts for over 90% of total diabetes cases worldwide, with its pathogenesis primarily attributed to modifiable lifestyle-related factors rather than genetic predisposition [3]. At present, lifestyle changes and drug therapy are the only approaches to treat type 2 diabetes [4]; however, diabetes control and treatment mainly rely on Western medicine, yet most of these drugs have been demonstrated to exhibit notable toxicity and adverse effects, especially α-glucosidase inhibitors (such as Acarbose and Voglibose), which can cause adverse digestive system reactions and hepatotoxicity. Metformin and GLP-1 receptor agonists (including liraglutide, semaglutide, and exenatide) all induce gastrointestinal adverse effects like vomiting, diarrhea, anorexia, and constipation [5]. Thus, there is a pressing need to uncover safer, more effective therapeutic strategies or drugs to address the current dilemma of “taking medicine is more uncomfortable”. Polysaccharides demonstrate pharmacological potential in glucose homeostasis modulation due to their natural and non-toxic properties [6,7].

Naturally prevalent biomacromolecules, polysaccharides, display diverse characteristics and fulfill essential biological roles across organisms [8]. These include antioxidant, immunomodulatory, anti-tumor, anti-hypertensive, anti-inflammatory, antibacterial, and antidiabetic effects [9,10,11,12,13]. Emerging evidence elucidates polysaccharides’ multi-target antidiabetic pharmacodynamics, encompassing both metabolic regulation (glucose/lipid metabolism) and enzymatic interference (inhibition of carbohydrate-digesting enzymes), regulation of gut microbes, modulation of the gut microbiota-Short-Chain Fatty Acids (SCFAs)-GPRs (G-Protein-Coupled Receptors) axis, and activation of the PI3K (Phosphatidylinositol 3-Kinase)/AKT (Protein Kinase B) signaling pathway [14,15,16,17,18]. Polysaccharides can also promote intestinal peristalsis, accelerate the forward movement and elimination of intestinal contents and feces, and further positively impact the treatment of diabetes [7,19]. As previous studies have shown, Muscat Hamburg grape skin polysaccharide, with a relatively high glucuronic acid content, exerts a strong inhibitory effect on α-amylase and α-glucosidase [19]. It is worth studying its hypoglycemic mechanism. Nowadays, grape seed polyphenols and anthocyanins are the primary research directions in grape studies. Polysaccharides in grapes are often completely ignored, although they have great potential for exploration [20,21]. Therefore, studying Muscat Hamburg grape skin polysaccharides in the context of diabetes could facilitate more comprehensive development and utilization of grapes and promote the development of the grape industry.

Gut microbiota is a multifaceted system that contains a diversity of microbial species and exists in the human gut. It establishes a mutualistic association with the host organism and performs a pivotal function in the digestive and immune processes. Accumulating studies have confirmed the pivotal involvement of intestinal microbiota in modulating the pathogenesis and disease trajectory of type 2 diabetes mellitus [22,23,24]. A marked correlation has been identified between intestinal homeostasis and the insulin resistance index. It was said that gut microbiota has a relationship with the recovery of T2D through regulating the constitution of intestinal flora and enhancing the biosynthesis of SCFAs [25]. Although polysaccharides pose challenges for direct breakdown and utilization by the digestive system, they are a source of nutrition for gut microbiota. Gut microbiota can produce beneficial metabolites such as SCFAs via fermentation of polysaccharides [26]. These products not only affect signaling pathways in the gut but also influence tissues and organs outside the gut via blood circulation and metabolic pathways. Therefore, exploring the action mechanisms of polysaccharides is deemed to offer substantial benefits through the integration of gut microbiota and metabolomics.

This research protocol aims to elucidate the therapeutic mechanisms of GSP on glucometabolic dysregulation and gut microbiota composition in type 2 diabetic (T2D) rats induced by a high-fat diet combined with STZ reagent, which have been intragastrically administered with different doses of GSP for 30 days, assess its hypoglycemic activity, and explore the relationships between polysaccharide, host and microbiota by combining 16S rRNA sequencing with LC-MS/MS-based metabolomic profiling.

## 2. Materials and Methods

### 2.1. Materials

Muscat Hamburg grapes were collected from Bei Yuan Chun Market (Urumqi, Xinjiang, China). The extraction method and structural characterization (monosaccharide composition: glucose 43.80%, mannose 14.43%, arabinose 14.32%, galactose 11.82%, glucuronic acid 6.36%, galacturonic acid 3.15%, rhamnose 2.56%, xylose 1.36%, ribose 0.56%, and fucose 0.38%) of the grape skin polysaccharide (GSP) used in this study have been reported in a previous study [19]. To ensure consistency with the prior study, GSP was verified before the experiment, and the results were consistent with the published data. Streptozotocin (STZ) was purchased from Shanghai Macklin Biochemical Co., Ltd. (Shanghai, China); triglycerides (TG, S03027), total cholesterol (TC, S03042), high-density lipoprotein cholesterol (HDL–C, S03025), low-density lipoprotein cholesterol (LDL–C, S03029), superoxide dismutase (SOD, G4306), malondialdehyde (MDA, G4302), and glutathione peroxidase (GSH–Px, G4310) and assay kits including ELISA kits of serum insulin and hepatic glycogen were determined using assay kits from Wuhan Service Biotechnology Co., Ltd. (Wuhan, China). All other reagents were purchased from Shanghai Sangon Biotech Company Co., Ltd. (Shanghai, China) and Wuhan Service Biotechnology Co., Ltd., China. All utilized chemicals met analytical grade standards.

### 2.2. Animals and Experimental Design

Sixty 28-day-old male Sprague-Dawley (SD) rats, averaging 180 g in weight, were sourced from Xinjiang Medical University (Xinjiang, China). The rats were maintained in a controlled environment (25 °C, regulated humidity) with a 12:12 light-dark cycle for a 5-day acclimatization period. Following this, they were randomly allocated to different groups and designated as the NC group (normal control, n = 8) and model group (n = 52). The NC group was provided with a normocaloric reference diet, while the remaining rats received a high-fat diet over a period of four weeks.

**Figure 1:**
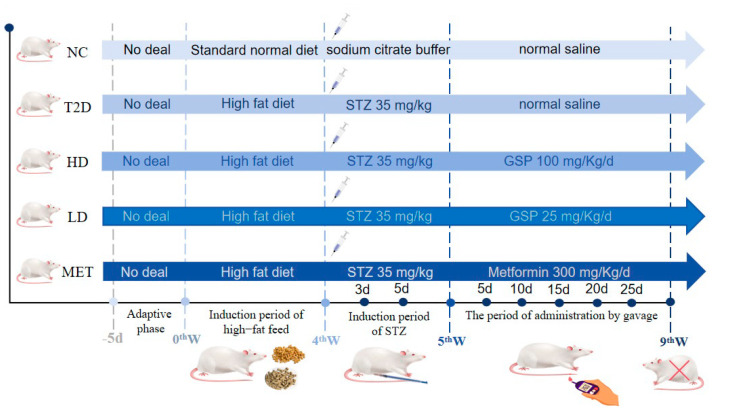
Model establishment. Normal control group (NC); Diabetes model group (T2D); GSP high-dose intragastric group (HD); GSP low-dose intragastric group (LD); Metformin gavage group (MET).

The T2D model was established in Figure 1 in accordance with Liu et al. [27] with modifications. Standard normal diet and high-fat diet were bought from Jiangsu Medicience Biomedical Co., Ltd. (Yangzhou, China), and the high-fat diet was composed of 54% rodent maintenance feed, 10% sucrose, 6% whole milk powder, 10% soy protein isolate, and 20% lard. Following a four-week induction period, the model group received intraperitoneal STZ (35 mg/kg) injections using 0.1 M sodium citrate buffer (pH 4.4) as the solvent, while NC rats were provided with an equivalent volume of 0.1 M citrate buffer. FBG was measured on the 3rd and 7th days after the injection period, and modeling was considered successful if FBG ≥ 11.1 mmol/L. If modeling was unsuccessful, the above steps were repeated three days later. On the third and last day of the induction week, fasting blood glucose (FBG) of each rat was monitored after fasting for 12 h. Rats with FBG greater than 11.1 mmol/L in both measurements were considered successfully established T2D models. Meanwhile, the model group was randomized into four cohorts as follows: T2D group (untreated, n = 13), HD group (GSP 100 mg/kg, n = 13), LD group (GSP 25 mg/kg, n = 13), and MET group (metformin 300 mg/kg, n = 13). The polysaccharide-treated group (HD and LD groups) and metformin group (MET group) were gavaged with the designed concentrations, while the NC group and T2D group were gavaged with equal volumes of normal saline (NS). This animal experimental procedure was rigorously performed following the operational guidelines of the Animals (Scientific Procedures) Act 1986, its associated regulations, and the EU Directive 2010/63 on the protection of animals used in scientific procedures. In addition, the experimental protocol gained clearance from the Institutional Animal Care and Use Committee (IACUC) of Xinjiang Agricultural University (Permit No. SCXK (Xin) 2023-0001) following formal review.

### 2.3. Collection of Samples

Following a 4-week oral administration period, all rats were fasted overnight. After anesthesia with 3% sodium pentobarbital via intraperitoneal injection, blood was sampled from the abdominal aorta. The obtained blood was placed in the refrigerator for 30 min to allow natural sedimentation, after which the blood was centrifuged at 2500× *g* for 15 min and stored at −80 °C. The liver, kidneys, and cecum were cut off, rinsed with normal saline, and weighed, respectively. Then the liver was divided into two parts. One part, together with the kidneys and cecum, was placed in the tissue fixative solution, and the other part was stored at −80 °C for subsequent work. Rat fecal samples were collected from the cecum, transferred to sterile cryopreservation tubes, and immediately stored at −80 °C for subsequent gut microbiota sequencing and metabolite determination.

### 2.4. Body Weights, FBG, HOMA-IR, HOMA-β, InS, and OGTT

After an overnight fast, FBG was measured using a standard glucometer via tail vein puncture. FBG (fasting blood glucose) and body weights were measured and recorded to monitor the changes in basic indicators of rats every five days. Upon completion of the study, an OGTT (oral glucose tolerance test) was conducted following a 12 h food deprivation period [28]. Similarly to the previous procedure, blood glucose concentrations were assessed at five defined time points (0, 0.5, 1.0, 1.5, and 2 h) following oral administration of a 20% glucose solution (2 g/kg) in each rat. Serum insulin (InS) concentrations were quantified via a commercial ELISA kit. Insulin resistance index (HOMA-IR), insulin sensitivity index (ISI), and insulin secretion index (HOMA-β) were determined using the formulas:

(1) HOMA-IR = INS × FGB/22.5

(2) $$\mathrm{ISI}={\mathrm{Ln}(\mathrm{FBG}\;\times \;\mathrm{InS})}^{-1}$$

(3) HOMA-β=20 × InS/(FBG - 3.5)

### 2.5. Assessment of Hepatic Oxidative Stress and Glycogen Levels

The levels of SOD, MDA, GSH–Px, and glycogen in the liver were measured per the methodology in the producer’s guideline.

### 2.6. Serum Biochemistry

The concentrations of TG (triglycerides), TC (total cholesterol), LDL–C (low-density lipoprotein), and HDL–C (high-density lipoprotein) in rat serum were determined using assay kits following the specific instructions outlined per the methodology in the manufacturer’s guidelines.

### 2.7. Histopathological Analysis of Kidney, Liver, and Intestine

Liver, intestinal, and kidney tissues underwent formalin fixation, dehydration, paraffin embedding, and sectioning. These sections were subjected to staining with hematoxylin and eosin to prepare H&E-stained slides. Pathological changes in these tissues were systematically examined under a light microscope.

### 2.8. Gut Microbiota Analysis

16S rRNA amplicon sequencing was carried out by Genesky Biotechnologies Inc. (Shanghai, China). In brief, total genomic DNA was extracted via the FastDNA SPIN Kit for Soil (MP Biomedicals, Santa Ana, CA, USA) in accordance with the producer’s guidelines. DNA integrity was assessed by 1% agarose gel electrophoresis, where concentration and purity were measured using a NanoDrop 2000 spectrophotometer (Thermo Fisher Scientific, Waltham, MA, USA) and further quantified, respectively, with a Qubit 3.0 Fluorometer (Invitrogen, Carlsbad, CA, USA). The V3–V4 hypervariable regions of the bacterial 16S rRNA gene were amplified with primers 341F (5′-CCTACGGGNGGCWGCAG-3′) and 805R (5′-GACTACHVGGGTATCTAATCC-3′). PCR amplicons were purified and sequenced using the Illumina NovaSeq 6000 platform (Illumina, Carlsbad, CA, USA) with 250 bp paired-end reads. Raw read sequences were analyzed using QIIME2 [29]. Adapter and primer sequences were removed via the cutadapt plugin. The DADA2 plugin was employed to perform quality control and identify amplicon sequence variants (ASVs) [30]. Taxonomic classification of ASV representative sequences was conducted via a pre-trained Naïve Bayes classifier (confidence threshold ≥ 0.7) trained on the SILVA ribosomal RNA database (release 138.2).

### 2.9. SCFAs Analysis

Briefly, colonic contents for each experimental animal were homogenized in deionized water using a 1:4.5 (*w*/*v*) proportion. Following initial preparation, all suspensions were subjected to thorough vortex mixing followed by 10 min of ultrasonic treatment. Subsequently, the processed colonic contents were immersed in an ice-water bath for 20 min, and then the supernatant was collected via centrifugation of the sample at 4800× *g* for 20 min at 4 °C. The procedure was repeated, and the supernatants combined. For SCFA analysis, the combined supernatant was analyzed by gas chromatography using an Agilent 7890A GC system (Agilent Technologies, Santa Clara, CA, USA) equipped with a DB-FFAP capillary column (30 m × 0.25 mm × 0.25 μm; J&W Scientific, Folsom, CA, USA). The injector temperature was set at 250 °C, and nitrogen was used as the carrier gas with a constant flow rate of 1.0 mL/min. 2-Ethylbutyric acid was employed as the internal standard. SCFAs were ultimately conducted through gas chromatography [27].

### 2.10. Cecal Content Metabolites (Untargeted Metabolomics Analysis)

Metabolomic profiling was performed via a Thermo Vanquish ultra-high performance liquid chromatography (UHPLC) system integrated with an ACQUITY UPLC^®^ HSS T3 analytical column (2.1 × 100 mm, 1.8 μm particle size; Waters Corporation, Milford, CT, USA). Chromatographic separation was performed under thermostatically controlled conditions (40 °C) with a mobile phase flow rate optimized for compound resolution [27]. Chromatographic separation parameters were standardized at a flow rate of 0.3 mL/min with 2 μL injection aliquots. In positive ionization mode [LC-ESI(+)-MS], the mobile phase was composed of acetonitrile containing 0.1% formic acid and aqueous formic acid solution. Conversely, negative ionization mode [LC-ESI(−)-MS] employed a buffered salt system combining acetonitrile and ammonium formate (5 mM) [31].

Metabolite profiling was conducted on an Orbitrap Exploris 120 mass spectrometer (Thermo Fisher Scientific, USA) fitted with electrospray ionization (ESI). Operational parameters were set to sheath/auxiliary gas flows of 40 and 10 arb, respectively, with ion transfer capillary temperature maintained at 325 °C. Spray voltage was set at 3.50 kV in positive ion mode and −2.50 kV in negative ion mode. Full-scan mass spectra were acquired at 60,000 resolution (*m*/*z* 200), and MS/MS spectra were recorded at 15,000 resolution targeting the top-four most intense precursor ions, using a dynamic exclusion algorithm to minimize redundant fragmentation events.

### 2.11. Statistical Analysis

Statistical processing was executed via one-way ANOVA using SPSS 17.0 (SPSS Inc., Chicago, IL, USA), with intergroup variations assessed through Tukey’s post hoc comparisons. Experimental outcomes were quantified as mean ± SD, adopting a significance threshold of *p* < 0.05 for statistical interpretation.

## 3. Results

### 3.1. Influence of GSP on Diabetes 2-Associated Indicators

**Figure 2:**
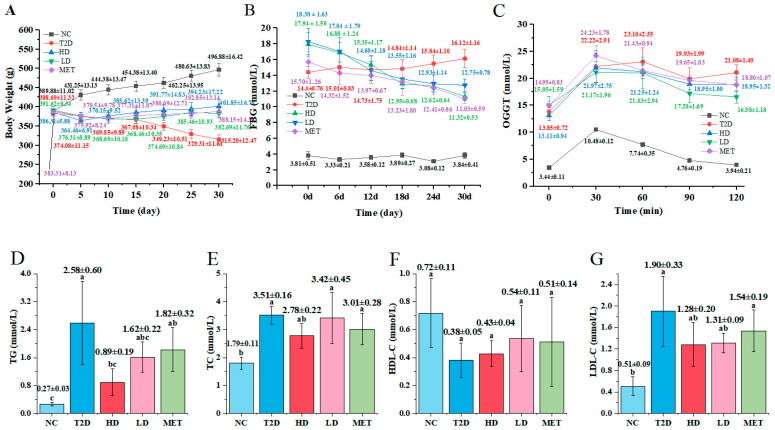
Effects of GSP in T2D rats: body weight (**A**), FBG (**B**), OGTT (**C**), TG (**D**), TC (**E**), HDL–C (**F**), and LDL–C (**G**). Data are expressed as the mean ± SD (n = 5); different superscript letters indicate statistically significant differences among groups (*p* < 0.05). Identical letters denote no significant difference.

Subsequent to GSP-metformin intervention, changes in body weight were monitored (as shown in Figure 2A). At the beginning of the intervention, body weight remained different across all experimental groups. Following model induction, the body weight of T2D rats was significantly lower than that of the normal control group, indicating obvious weight loss. The NC group exhibited a steady rise in body weight during the administration period, whereas the T2D group’s weight declined from 388.69 g ± 11.32 g to 315.23 g ± 12.47 g (decreased by approximately 23.30%) over the 30-day period. Compared to the T2D group, treatment with GSP and metformin via oral administration ameliorated the body weight of diabetic rats to varying extents. From day 5 to 30, the HD group’s weight rose from 364.46 ± 6.91 g to 401.85 ± 16.79 g (increased by approximately 10.26%), while in the LD group, it increased from 376.31 ± 8.89 g to 382.69 ± 11.76 g (increased by approximately 1.70%). These results suggest that GSP exhibits the potential to mitigate weight loss symptoms in diabetic rats.

Figure 2 and Table 1 summarize the impacts of GSP and metformin on diabetic parameters in T2D rats, including FBG, OGTT, ISI, HOMA-IR, and HOMA-β. FBG concentrations were monitored at five-day intervals across experimental cohorts (Figure 2B). In the NC group, FBG levels remained stable (around 3.09–3.84 mmol/L). In contrast, the T2D group exhibited the highest FBG levels (14.4–16.12 mmol/L), with no spontaneous reduction in FBG levels observed from model establishment to the end of the intervention. These findings confirmed the successful induction of the T2D rat model [32]. Following 30 days of oral administration of GSP and metformin, significant decreases were observed in MET, HD, and LD (15.70–11.03 mmol/L, 17.94–11.32 mmol/L, and 18.30–12.75 mmol/L, respectively) groups.

Following the oral administration of a 2.0 g/kg glucose solution, blood glucose levels were assessed at 30-min intervals from 0 to 120 min (as shown in Figure 2C). All five groups exhibited a similar trend: blood glucose levels peaked approximately 30 min post-administration and then slowly declined back to the original levels. The NC group maintained stable glucose levels (3.44–10.48 mmol/L). Conversely, the T2D group exhibited a significant increase in blood glucose, reaching significantly higher values (13.85–22.22 mmol/L). Compared to the T2D group, the treatment groups (HD, LD, and MET) demonstrated a decrease in blood glucose levels. Specifically, after 30 min, blood glucose in the HD, LD, and MET groups dropped rapidly (from 21.97 to 18.95 mmol/L, 21.17 to 16.58 mmol/L, and 24.23 to 18.80 mmol/L, respectively), though these levels remained significantly higher than those in the NC group. Concurrently, the T2D group displayed markedly decreased ISI and HOMA-β (Table 1), suggesting that the high-fat feeding regimen significantly disrupted glycemic homeostasis, manifested as impaired insulin sensitivity and aberrant glucose regulation [32]. Therapeutic interventions demonstrated enhanced ISI concomitant with reduced HOMA-IR values relative to model controls, indicating that GSP significantly improved insulin resistance and promoted insulin utilization in T2D rats.

### 3.2. Influence of GSP on Serum Lipids in Rats with Type 2 Diabetes

Dysregulated lipid metabolism is a key pathological feature of diabetes [33,34]. Elevated serum levels of TC and TG (key indicators of diabetes progression) are primarily attributed to impaired insulin regulation [35]. Concurrently, the activity of hepatic lipase decreases with the dysfunction of pancreatic islet function, resulting in reduced production of HDL–C and elevated LDL–C levels in the liver and blood [36]. As shown in Figure 2, the T2D group exhibited severe dyslipidemia with respect to the NC group: serum TG increased by 158.30% (Figure 2D), TC by 179.23% (Figure 2E), LDL–C by 189.90% (Figure 2G), and HDL–C decreased by 38.18% (Figure 2F). After 30 days of treatment, TG, TC, and LDL–C levels declined, while HDL–C levels showed an upward trend. These improvements were most pronounced in the GSP treatments and the MET group.

### 3.3. Influence of GSP on Hepatic Oxidative Stress and Glycogen in T2D Rats

**Figure 3:**
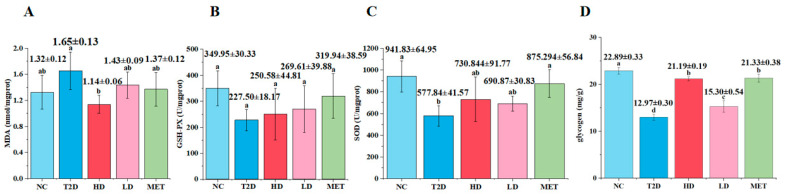
Effects of GSP on the oxidative status of the liver in T2D rats: MDA (**A**), GSH–Px (**B**), SOD (**C**), and the glycogen of the liver (**D**). Data are expressed as the mean ± SD (n = 5); different superscript letters indicate statistically significant differences among groups (*p* < 0.05). Identical letters denote no significant difference.

Oxidative stress plays a crucial role in the onset and development of diabetes mellitus [37]. The hepatic oxidative stress in T2D rats was reflected by measuring MDA content, GSH–Px activity, and SOD activity (illustrated in Figure 3). In relation to the NC group, the model group displayed a marked elevation in MDA content along with significant reductions in GSH–Px activity, SOD activity, and glycogen levels. Following administration of GSP and metformin, diabetic rats exhibited elevated GSH–Px activity, SOD activity, and glycogen concentrations, with more marked increases observed in the HD (high-dose GSP) and MET (metformin monotherapy) groups. These findings suggest that GSP and metformin administration ameliorate hepatic oxidative stress in diabetic rats.

### 3.4. Microscopic Examination of Tissue Structures

**Figure 4:**
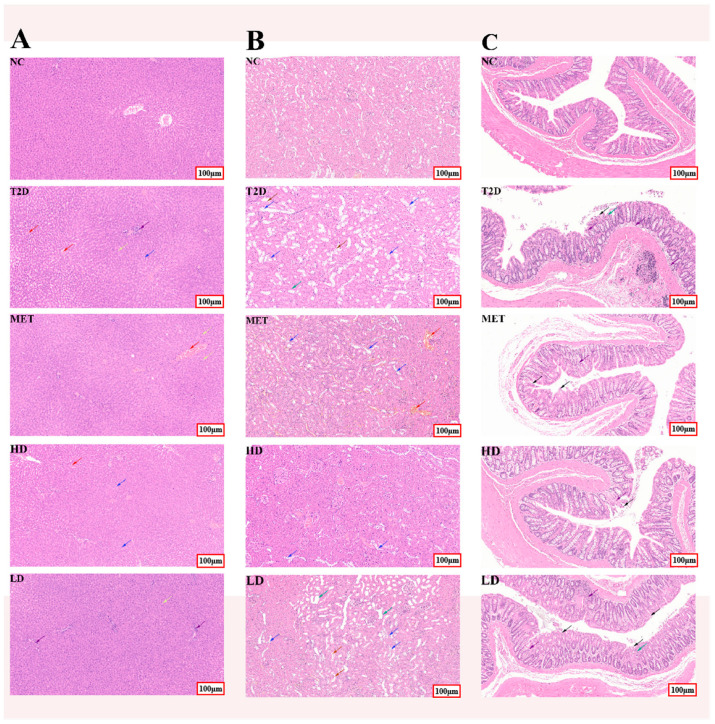
Histopathological analysis of liver (**A**), kidney (**B**), and intestine (**C**) in rats from five groups was conducted via hematoxylin and eosin staining (magnification, 100×).

Histopathological analysis of the liver, kidney, and intestine in rats across five groups is illustrated in Figure 4. The histological scoring criteria were adapted with minor modifications from Kihara [38], Tang [39], and Wang [40], and the detailed adjusted criteria are presented in Table 2. Figure 4A revealed that liver cells in the NC group displayed normal, round, and plump morphology with no inflammatory cell infiltration. In contrast, the cells in the liver tissue of rats from the T2D group exhibited an edematous state (blue arrow). The cytoplasm was loosely structured and lightly stained, with round vacuoles observable internally, alongside a small quantity of fatty degeneration in cells (yellow arrow) and the appearance of inflammatory cells (purple arrow). With the addition of GSP treatments, these pathological features were alleviated. Notably, the recovery effect of high-dose GSP was a more pronounced recovery effect, and inflammatory cells disappeared in the HD group. The recovery effect of the HD group was comparable to that of the MET group. Kidney sections of the rat in the NC group exhibited normal morphological features (Figure 4B). In contrast, for the rat in the T2D group, numerous renal tubular epithelial cells presented edema with cellular swelling (blue arrow), and their cytoplasm was loosely structured, lightly stained, or vacuolar. A small number of renal tubules contained eosinophilic substances and detached epithelial cells (brown arrow); some renal tubules showed dilation with luminal dilation and deformation (green arrow), accompanied by flattening of renal tubular epithelial cells. And the red arrows indicate congestion. After the intervention of GSPs and metformin, the shape of renal tubules tended to be normal. Figure 4C showed that the intestines of rats from the NC group showed normal morphological features. The intestinal glands in the lamina propria were densely packed and tubular, and goblet cells were abundant, with no obvious abnormalities observed in the intestinal structure. Conversely, in the T2D group, substantial mucosal epithelial cell shedding was observed (black arrow); the lamina propria showed disordered arrangement of intestinal glands, reduced goblet cells, and mild connective tissue hyperplasia (green arrow), and a considerable level of inflammatory cell infiltration, mainly composed of lymphocytes, can be observed (purple arrow). With the development of interference, the phenomena of mucosal epithelial shedding and lymphocyte infiltration were alleviated to varying extents in the MET, HD, and LD groups.

### 3.5. Influence of GSP on the Intestinal Microbiota of Diabetes 2-Associated

**Figure 5:**
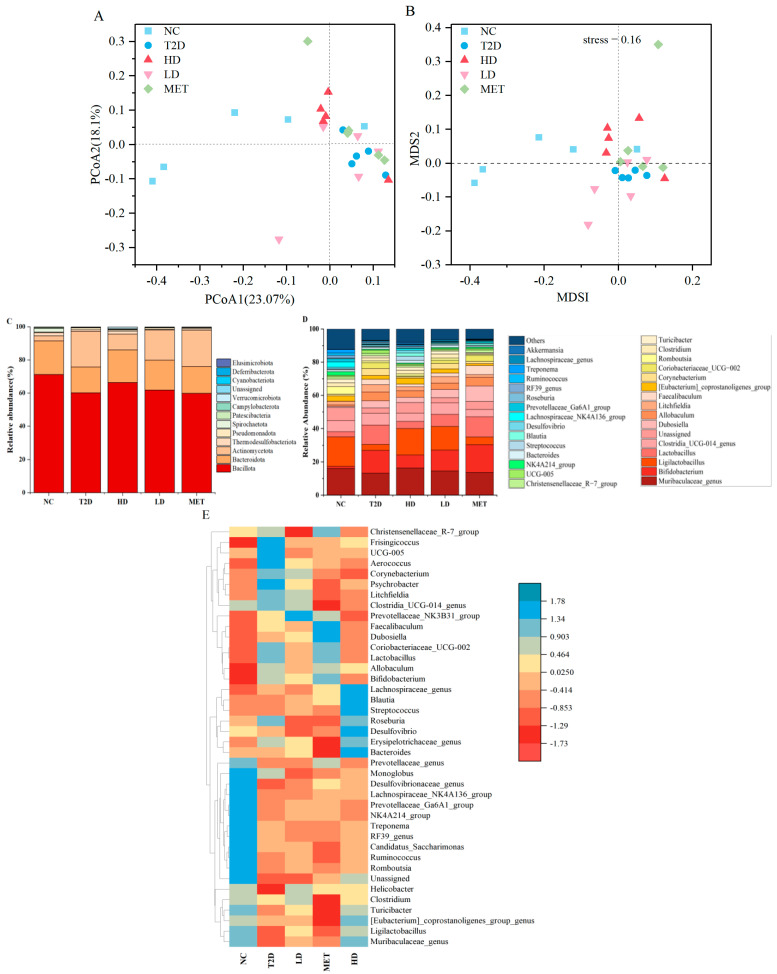
GSP-mediated effects on gut microbiota in T2D rats at the phylum (**A**) and genus (**B**) taxonomic levels; genus-level heatmap (**C**); PCoA analysis using weighted UniFrac distance (**D**); and NMDS analysis (**E**) across five groups (n = 4).

The impacts of different doses of GSP on the constituent profile of gut microbiota in T2D rats were analyzed via 16S rRNA sequencing. For α-diversity, coverage indices were close to 1, which indicates that sequences in samples were fully detected and the sequencing results were reliable and valid. Chao1 and ACE indices revealed that the NC group exhibited higher microbial richness compared to the T2D group, as presented in Table 3. During the intervention period, microbial richness (Chao1/ACE indices) gradually increased in the MET, LD, and HD groups. What is more, the HD group shows the most pronounced improvement. For β-diversity, community structure was evaluated by principal component analysis (PCA). As depicted in Figure 5A,B, microbial composition in the NC group exhibited marked separation from that of the T2D group, indicating distinct variations in microbiota composition were observed among the groups. Notably, Figure 5A,B revealed discernible divergence of the HD cohort from the T2D subjects and partial overlap with the NC controls, demonstrating GSP’s regulatory ability to restore gut microbiota toward a healthier state.

A multi-level taxonomic analysis was conducted to characterize microbial architecture across groups. As pictured in Figure 5C, at the phylum taxonomic level, the intestinal flora of all the groups was composed primarily of Bacillota, Bacteroidetes, and Actinobacteria [27]. When contrasted with the NC group, the T2D group displayed a marked decline in the relative abundances of Bacillota and Bacteroidetes, with a significant rise observed in Actinobacteria’s relative abundance. This observation corroborates previous findings that high-fat diets reduce the relative abundance of Bacteroidetes at the phylum level [27]. The relative abundance of Actinobacteria decreased in the treatment groups, and the abundance of Bacillota and Bacteroidetes increased during the 30 days of treatment. Elevated Actinobacteria abundance is associated with gut homeostasis and metabolic dysregulation [41]. Additionally, the relative abundance of Thermodesulfobacteriota, Deferribacterota, and Cyanobacteriota showed a significant reduction in T2D. The proportion of Bifidobacterium and Lactobacillus increased rapidly in T2D rats, which in the HD group was between NC and T2D (Figure 5D). The metabolic predominance of dominant genera (Bifidobacterium/Lactobacillus) likely originated from their glycolytic proficiency, enabling efficient energy harvest in carbohydrate-rich milieus [27,42]. In fact, Bifidobacterium and Lactobacillus are integral parts of human health, which have documented efficacy in alleviating the symptoms of diabetes [43]. With the intervention of GSP, the symptoms of T2D were in remission, and there was stabilization in the intestinal ecology. Following commensal microbial reconstitution, the competitive dominance of Bifidobacterium and Lactobacillus diminished concomitantly with their suppressed proliferation. Additionally, the increase in butyrate-producing Roseburia and metabolically versatile Blautia meant that the intake of GSP effectively developed the intestinal homeostasis in T2D rats. Heatmap analysis additionally revealed a comparable trend; in the HD group, Blautia and Roseburia, which were colored in blue, scored higher (Figure 5E). The color intensity of Bifidobacterium and Lactobacillus ranged between red and blue, which meant the proportion of Bifidobacterium and Lactobacillus in HD was between NC and T2D.

### 3.6. Influence of GSP on Diabetes 2-Associated SCFAs

As established, polysaccharides are hard to digest in the gut. However, they can be fermented by the gut microbiota and processed into SCFAs [44]. It was said that the most effective way of the therapeutic effect of polysaccharides on diabetes is mediated by the intestinal SCFA levels. Table 4 shows the influences of GSP on intestinal SCFAs, including acetic acid, propionic acid, and butyric acid. Relative to the NC cohort, these acids decreased in the T2D group in degrees. With the treatment of GSP and metformin, the decrease in the SCFAs above was reversed. The concentrations of propionic acid and butyric acid in the HD group were even higher than those in the NC group, indicating that GSP had the ability to modulate the gut microenvironment via SCFA production.

### 3.7. Influence of GSP on Diabetes 2-Associated Metabolic Products

**Figure 6:**
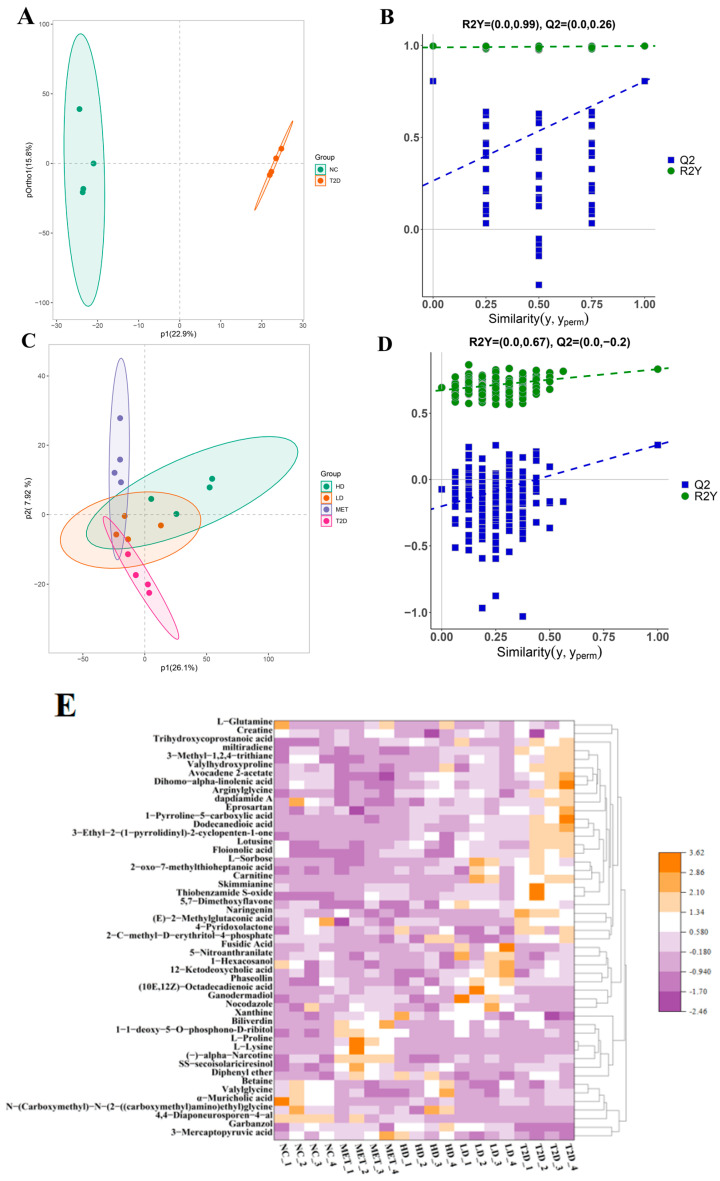

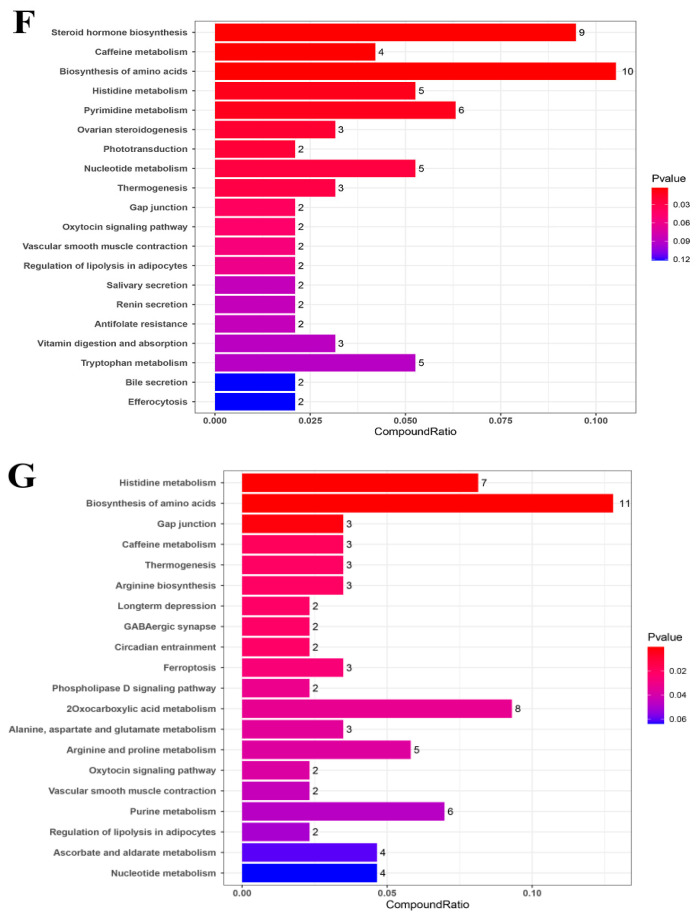
Untargeted metabolomic profiling: OPLS-DA comparison between the NC and T2D groups (**A**), the permutation test diagram of the OPLS-DA model between NC and T2D (**B**), OPLS-DA analysis between HD and LD vs. MET and T2D (**C**), the permutation test diagram of the OPLS-DA model between HD and LD vs. MET and T2D (**D**), heatmap of different metabolites in feces among NC, T2D, HD, LD, and MET groups (**E**), bar chart of influencing factors of metabolic pathways between NC and T2D (**F**), and bar chart of influencing factors of metabolic pathways between NC and T2D vs. HD (**G**).

Polysaccharides are fermented into intestinal small-molecule metabolites that regulate intestinal function. To comprehensively explore the mechanisms underlying GSP intervention in T2D rats, a non-targeted metabolomics approach was studied. Orthogonal partial least-squares discriminant analysis (OPLS-DA) modeling demonstrated obvious differences in metabolomic profiles between NC and T2D cohorts (Figure 6A), confirming significant intergroup disparities at the metabolomic level. The model was validated by permutation. In Figure 6C, the HD, LD, and MET groups had a little overlapping area, while most of them were separated from each other. This phenomenon means that GSP and MET groups have different metabolomics. Moreover, there may be differences in treatment mechanisms. From Figure 6B,D, all blue Q^2^ points (from permutation tests) are lower from left to right compared to the original Q^2^ point (on the far right). Additionally, the blue Q^2^ regression line displayed a negative intercept, showing that the model results are reliable and valid [35].

Variable importance to projection (VIP) >1 and *p* < 0.05 were used to screen discriminatory metabolites related to diabetes. As illustrated in Figure 6E, GSP and metformin interventions significantly modulated 50 metabolites across five experimental groups. These included amino acids, bile acids and their biosynthetic intermediates, short-chain fatty acid derivatives, and redox-related compounds. These alterations potentially regulate host glucose-lipid metabolism and oxidative stress responses [35,45]. Additionally, higher levels of betaine, xanthine, carnitine, glutamine, and glycine were regarded as a marker of lower T2D risk, and higher creatine was regarded as a marker of higher T2D risk [46,47]. In this study, GSP increased betaine and valylglycine levels, kept the levels of L-glutamine, biliverdin, and xanthine stable, and reduced creatine. Metformin differed from GSP. It seems that metformin lowers blood sugar by regulating amino acids (L-proline, L-lysine), xanthine, α-narcotine, SS-secoisolariciresinol, biliverdin, and diphenyl ether. Notably, different GSP doses had different effects. 5-nitroanthranilate, 1-hexacosanol, 12-ketodeoxycholic acid, phaseollin, (10E,12Z)-octadecadienoic acid, ganodermadiol, and xanthine were significantly increased in the LD group, which were related to antibacterial activity, anti-inflammatory effects, and bile acid metabolism.

As depicted in Figure 6F, marked differences in metabolic pathways were identified between the NC and T2D groups, particularly in steroid hormone biosynthesis, biosynthesis of amino acids, histidine metabolism, pyrimidine metabolism, ovarian steroidogenesis, nucleotide metabolism, and bile secretion. These pathways are closely linked to steroid-related substances, amino acid synthesis, bile acid metabolism, energy homeostasis, and drug metabolism [27], which collectively suggest severe metabolic dysregulation in diabetic rats characterized by multiple aberrant biological processes.

From Figure 6G, it can be observed that metabolic pathway alterations occurred in the HD group. These differences were mainly proved by the reduction in steroid-related substances and bile acids, the increase in the kinds of amino acid metabolic pathways (alanine, aspartate, proline, arginine, and glutamate metabolism), the sudden appearance in ascorbate and aldarate metabolism, and the increase in intercellular signal transduction and cell division, suggesting that the mechanism of GSP-H treating T2D is related to the above substances. This is further supported by the changing trends of metabolites such as trihydroxycoprostanoic acid (↓0.87-fold, *p* < 0.01), α-muricholic acid (↑0.29-fold, *p* < 0.05), L-proline (↓0.11-fold, *p* < 0.05), 1-Pyrroline-5-carboxylic acid (↓1.07-fold, *p* < 0.01), Arginylglycine (↓0.70-fold, *p* < 0.01), (E)-2-Methylglutaconic acid (↓1.12-fold, *p* < 0.01), and L-sorbose (↓1.52-fold, *p* < 0.01) in Figure 6E, which indicates that high GSP treatment exerts an impact on the metabolic pathways of T2D.

### 3.8. Spearman’s Correlation Analysis

**Figure 7:**
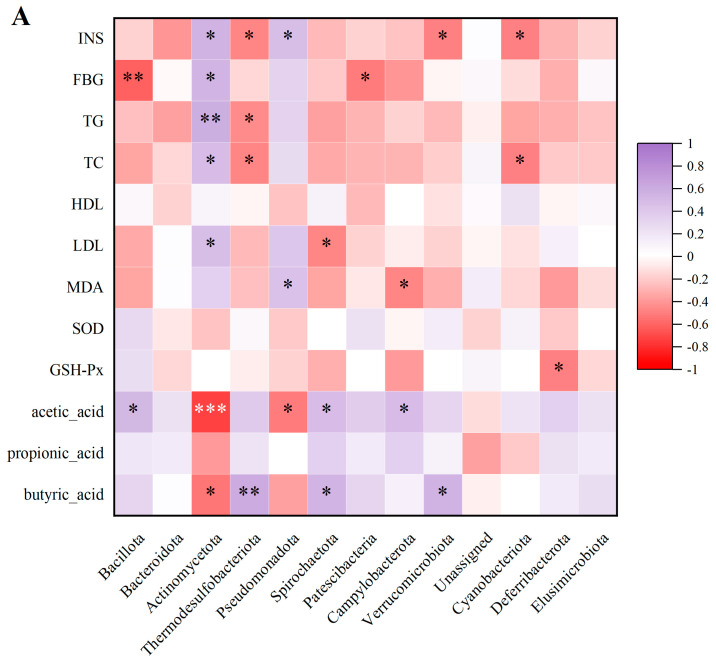

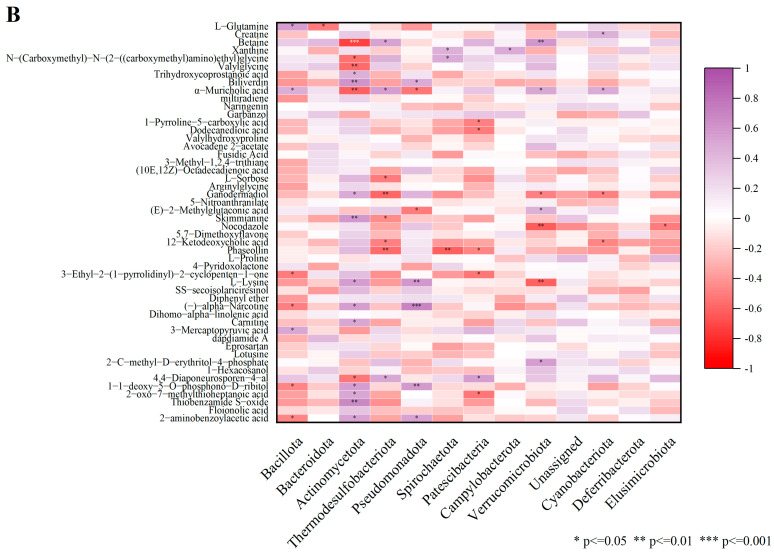
Spearman correlation heatmaps illustrating the associations between gut microbiota and biochemical parameters/SCFAs (**A**), as well as between gut microbiota and metabolites (**B**).

The evaluation of the correlation between some biochemical indexes and gut microbiota in rats was performed with Spearman’s correlation analysis. From Figure 7A, the abundance of Actinomycetota and Pseudomonadota exhibited positive correlations with INS, FBG, TG, TC, and LDL–C and negative associations with SCFAs, especially the alterations in acetic acid and butyric acid. Higher levels of Patescibacteria, Cyanobacteriota, and Deferribacterota were associated with reduced concentrations of INS, FBG, TC, and GSH–Px. The abundance of Thermodesulfobacteriota and Spirochaetota had positive associations with the changes in acetic acid and butyric acid and negative associations with the levels of INS, TG, TC, and LDL–C.

As demonstrated in Figure 7B, Actinomycetota, Thermodesulfobacteriota, and Pseudomonadota showed strong correlations with the metabolites. For instance, Actinomycetota had a positive relationship with 11 kinds of metabolites, including trihydroxycoprostanoic acid, biliverdin, skimmianine, and thiobenzamide S-oxide, and negative relationships with 5 kinds of metabolites, such as betaine, N,N-glycine, valylglycine, α-muricholic acid, and 4,4-diaponeurosporen-4-al. Thermodesulfobacteriota had an opposite performance. It had a positive relationship with betaine, α-muricholic acid, and 4,4-diaponeurosporen-4-al while having negative relationships with L-sorbose and skimmianine. Pseudomonadota had the same trend as Actinomycetota. As established, there exists a tight relationship between intestinal microorganisms and metabolic pathways. These findings suggested that GSP can regulate the gut microbiota and change their metabolites to influence metabolic pathways.

## 4. Discussion

The hallmark metabolic characteristics of T2D rats encompass reduced body weight, elevated FBG, abnormal glucose tolerance, insulin resistance, and hyperinsulinemia, all attributable to diabetes-associated energy metabolism dysregulation [48]. The absorption and utilization of nutrients (e.g., glucose, lipids) were affected by the hyperinsulinemia and insulin; to obtain more energy, the nutrients were expended more, and there was no nutrition to store [49]. Results revealed that the serum insulin levels were significantly higher in the T2D group compared with the NC group. After treatment, GSP and MET significantly reduced the hyperinsulinemia and HOMA-IR index, greatly alleviating insulin resistance in T2D rats, improving the utilization of nutrients, resulting in body weight gain, and reversing the increase in FBG and OGTT [50,51].

Decreasing the liver fat is a strategy to ameliorate the symptoms of T2D rats [52]. Under conditions of hyperglycemia, hepatocytes enhance glycogen synthesis and storage, while the occurrence of insulin resistance compromises the inhibition of glycogen breakdown, leading to increased glycogenolysis [6]. Prolonged hyperglycemia exacerbates the metabolic burden on the liver, resulting in disordered hepatic glycogen metabolism [53]. Therefore, liver glycogen and lipid metabolism were examined in this work. GSP not only ameliorated dyslipidemia by reducing serum TG, TC, and LDL–C levels and elevating HDL–C, but also attenuated hepatic glycogen accumulation [49,51,54].

In addition, the liver is the primary oxidative stress target organ in T2D rats [55]. It is invariably accompanied by disordered lipid metabolism, triggering heightened ROS (reactive oxygen species) generation, compromising redox homeostasis, and facilitating accumulation of lipid peroxidation byproducts [56]. In this work, following 30-day interventions with GSP and MET, hepatic antioxidant enzymes (SOD and GSH) were significantly elevated in T2D rats, whereas the lipid peroxidation marker MDA showed marked reduction. This antioxidant enhancement may be attributed to GSP’s unique monosaccharide profile, particularly its high glucuronic acid content, which exhibits potent free radical scavenging capacity [57].

An environment rich in sugar and fat often causes the histopathological damage of the liver, kidneys, and intestines by the overproduction of reactive oxygen species [58]. The morphological results showed that GSP and MET treatment groups ameliorated tissue damage in the liver, kidneys, and intestines, which may be mediated through the improvement of insulin resistance, relief of oxidative stress, and alterations in gut microbiota, as well as their associated metabolites.

Gut microbes are the secondary genetic reservoir of the human body and form an intricate symbiotic interaction with the host, exerting a critical function in preserving host homeostasis, regulating the immune system, and affecting metabolic diseases. Research indicates that intestinal microbial communities mitigate metabolic dysregulation in obesity and diabetes through modulation of caloric assimilation, redox homeostasis, and glycemic control [59,60,61]. In rats, over 90% of the intestinal microbiota comprises three dominant phyla: Bacillota, Bacteroidetes, and Actinobacteria. Concurrently, the levels of the first two usually decreased in diabetic patients [62,63]. However, GSP intervention restored their populations to normal levels and concurrently elevated SCFA production [35,64]. The HD group exhibited a higher level of related microorganisms, which could produce SCFAs like Lachnospira and others [65]. It was said that Bacteroides and Akkermansia could produce propionic acid directly or indirectly, and butyric acid was mainly produced by Roseburia, Eubacterium, and Fusobacterium [66]. The high levels of propionic acid and butyric acid in the HD group were further confirmed by higher concentrations of Bacteroides, Akkermansia, Roseburia, Eubacterium, and Fusobacterium. It was worth noting that the Muribaculaceae family could regulate the intestinal barrier function, inhibit inflammation, and exert a critical role in diabetes [67]. The observed intestinal histomorphological improvements in the HD group may be attributed to Muribaculaceae-mediated barrier enhancement.

SCFAs are mainly produced in the gut by the flora [68]. As established, SCFAs are well-characterized modulators of enteric homeostasis and caloric partitioning, exhibiting demonstrated efficacy in insulin receptor sensitization and glycemic regulation. Specifically, Propionate inhibits key lipogenic enzymes (acetyl-CoA carboxylase and fatty acid synthase), reduces hepatic triglyceride accumulation, and improves hepatic insulin sensitivity. Butyrate, a key energy source for colonic epithelial cells, enhances mucosal barrier integrity and reduces intestinal permeability, thereby limiting lipopolysaccharide translocation into the systemic circulation and alleviating low-grade chronic inflammation, alleviating T2D symptoms [69,70]. In our study, the GSP-treated groups exhibited increased acetic acid levels (HD: 271.848 ± 2.589 μg/mL; LD: 248.92 ± 4.748 μg/mL) and butyrate levels (HD group: 68.690 ± 0.610 μg/mL; LD group: 51.300 ± 1.827 μg/mL), accompanied by elevated hepatic glycogen content (HD group: 21.19 ± 0.19 mg/g; LD group: 15.30 ± 0.54 mg/g) and pathological improvements in both cecal and hepatic tissues. Furthermore, the enrichment of acidogenic bacteria, including Bacteroides, Ackermannia, Roseburia, Eubacillus, Lachnospira, and Fusobacterium, further confirms that GSP ameliorates T2D by enhancing the abundance of acidogenic bacteria and subsequently increasing SCFA levels.

From the metabolic pathway perspective, in comparison with the NC group, the significant differences in steroid hormone biosynthesis, nucleotide metabolism, oxytocin signaling pathway, and bile secretion in the intestines of rats in the HD group decreased. Steroid hormone biosynthesis may reduce insulin sensitivity, with elevated metabolites such as 12-ketooctadecanoic acid and α-muricholic acid potentially contributing to this effect, while nucleotide metabolism could influence processes like hepatic cell proliferation and repair. Additionally, the oxytocin signaling pathway might impair organ blood supply in diabetic rats by altering vascular function, whereas bile acids may facilitate intestinal fat breakdown and absorption, thereby reducing lipid accumulation. The reduction in the significant differences meant that GSP-H administration may mitigate metabolic disparities between the NC and T2D groups through multifaceted mechanisms. And the emergence of arginine and proline metabolism suggested that GSP-H was capable of modulating glucose and lipid metabolism in T2D rats via enhancing blood glucose homeostasis and alleviating insulin resistance [71]. Meanwhile, in Figure 6E, the HD group exhibited altered levels of betaine and valylglycine, L-glutamine, biliverdin, xanthine, and creatine, which were consistent with the observed changes in metabolic pathways and different from those in the MET group. Although low GSP shared some effects with high GSP, there were a few differences in the metabolites. Low GSP only increased biliverdin and xanthine levels. Thus, that may be the reason for the different effects between high GSP and low GSP.

## 5. Conclusions

In short, GSP was proven to have good hypoglycemic and hypolipidemic activities. In terms of apparent indicators, it can reduce FBG, improve OGTT results, and reduce body weight. In terms of insulin sensitivity, it reduces the INS concentration and HOMA-IR. It can also improve the blood lipid levels (TG, TC, HDL–C, LDL–C), regulate the oxidative stress (MDA, SOD, GSH–Px), and glycogen. Previous studies have shown that purified GSP exhibits a higher uronic acid content and stronger inhibitory effects on α-amylase and α-glucosidase. In the present study, GSP can increase the content of acidogenic bacteria (Bacteroides, Ackermannia, Roseburia, Eubacillus, Lachnospira, and Fusobacterium), thereby raising the content of short-chain fatty acids, reducing blood glucose, and alleviating insulin resistance. Thus, we hypothesize that the hypoglycemic effect of GSP in this study may be associated with its uronic acid content, though this hypothesis requires further structure-activity relationship experiments for validation. Furthermore, untargeted metabolomic analysis revealed that high GSP doses exhibited altered levels of betaine, valylglycine, L-glutamine, biliverdin, xanthine, and creatine, while low GSP doses were related to antibacterial activity, anti-inflammatory effects, and bile acid metabolism. GSP shows promise as a functional supplement for T2D in rodent studies, but its therapeutic potential in humans requires rigorous clinical and translational research to validate efficacy and safety, indicating it could be a potential functional ingredient with therapeutic potential for T2D.

## Figures and Tables

**Table 1:** Impacts of GSP administration on insulin, ISI, HOMA-IR, and HOMA-β levels in T2D rats.

Groups	InS(mU/L)	ISI	HOMA-IR	HOMA-β
NC	24.228 ± 1.911 ^b^	0.210 ± 0.004 ^a^	5.277 ± 0.499 ^c^	356.609 ± 28.546 ^a^
T2D	55.218 ± 2.096 ^a^	0.149 ± 0.001 ^a^	35.090 ± 1.161 ^a^	103.541 ± 8.855 ^b^
HD	33.076 ± 3.245 ^b^	0.167 ± 0.002 ^b^	18.081 ± 1.745 ^b^	81.965 ± 17.488 ^b^
LD	44.393 ± 2.995 ^a^	0.160 ± 0.003 ^bc^	24.093 ± 3.523 ^b^	109.101 ± 11.485 ^b^
MET	32.720 ± 2.354 ^b^	0.159 ± 0.007 ^bc^	24.743 ± 2.965 ^b^	53.302 ± 8.559 ^b^

Note: Values are presented as mean ± SD (n = 5); different superscript letters indicate statistically significant differences among groups (*p* < 0.05). Identical letters denote no significant difference.

**Table 2:** Histological scoring criteria.

Histopathological Scoring System for Liver Disease						
	Pathological Feature	Degree of Severity	Score	Group	Final Score = Score (Inflammatory Activity + Hepatocellular Injury + Sinusoidal Congestion + Portal Tract Lesion)	Final Practical Score
Inflammatory Activity	None	None	0	NC	0	Nothing abnormal
	Individual	Mild	1			
	Most	Moderate	2			
	Virtually all	Severe	3			
	All	Complete necrosis	4	T2D	4	Mild
Hepatocellular Injury	None	None	0			
	Individual (Hepatocellular Edema <5%)	Mild	1			
	Most (Hepatocellular Edema 5–30%)	Moderate	2			
	Virtually all (Hepatocellular Edema >30%)	Severe	3	HD	2	Mild
	All	Complete necrosis	4			
Sinusoidal Congestion	None	None	0			
	Individual (sinusoidal dilation <25%)	Mild	1			
	Most (sinusoidal dilation 25–50%)	Moderate	2	LD	3	Mild
	Virtually all (sinusoidal dilation >50%)	Severe	3			
	All	Complete necrosis	4			
Portal Tract Lesion	None	None	0			
	Individual	Mild	1	MET	2	Mild
	Most (inflammatory cell infiltration <1/3)	Moderate	2			
	Virtually all (inflammatory cell infiltration >1/3)	Severe	3			
	All	Complete necrosis	4			
**Renal Pathological Scoring Criteria**						
	**Pathological Feature**	**Degree of Severity**	**Score**	**Group**	**Final Score = Score (Tubular Epithelial Cell Injury or Necrosis + Vacuolation of Renal Tubular Epithelial Cells + Swelling of Renal Tubular Epithelial Cells)**	**Final Practical Score**
Tubular Epithelial Cell Injury or Necrosis	None	None	0	NC	0	Nothing abnormal
	Individual	Mild	1			
	Most	Moderate	2			
	Virtually all	Severe	3	T2D	9	Severe
	All	Complete necrosis	4			
Vacuolation of Renal Tubular Epithelial Cells	None	None	0			
	Individual	Mild	1	HD	2	Mild
	Most	Moderate	2			
	Virtually all	Severe	3			
	All	Complete necrosis	4	LD	4	Mild
Swelling of Renal Tubular Epithelial Cells	None	None	0			
	Individual	Mild	1			
	Most	Moderate	2	MET	2	Mild
	Virtually all	Severe	3			
	All	Complete necrosis	4			
**Pathological Scoring Criteria for Appendicitis**						
	**Pathological Feature**	**Degree of Severity**	**Score**	**Group**	**Final Score = Score (Inflammation Severity + Inflammation Extent + Crypt Damage)**	**Final Practical Score**
Inflammation severity	None	None	0	NC	0	Nothing abnormal detected
	Individual	Mild	1			
	Most	Moderate	2			
	Virtually all	Severe	3	LD MET NC	6	Moderate

Inflammation extent	None	None	0			
	Mucosa involvement	Mild	1	T2D HD LD	3	Mild
	Submucosa propria involvement	Moderate	2			
	Transmural involvement	Severe	3			
Crypt damage	None	None	0	LD	5	Moderate
	Basal 1/3 damage	Mild	1			
	Basal 2/3 damage	Moderate	2	MET	3	Mild
	Crypt lost; surface epithelium present	Severe	3			
	Crypt and surface epithelium lost	Complete necrosis	4			

The final pathological score is defined as follows: Mild: 0 < Final Score ≤ 4; Moderate: 5 ≤ Final Score ≤ 8; Severe: 9 ≤ Final Score ≤ 11; Endpoint: Complete necrosis: Final Score ≥ 12.

**Table 3:** Changes in alpha diversity in T2D rats.

Group	Coverage	Chao1	Observed	Shannon	Simpson
NC	0.99982	657.05 ± 66.63 ^a^	653.40 ± 66.10 ^a^	4.68 ± 0.35 ^a^	0.05 ± 0.02 ^a^
T2D	0.99986	424.57 ± 45.75 ^b^	422.40 ± 45.24 ^b^	4.03 ± 0.25 ^a^	0.07 ± 0.02 ^a^
HD	0.99989	525.14 ± 36.80 ^ab^	523.40 ± 36.93 ^ab^	4.35 ± 0.21 ^a^	0.05 ± 0.02 ^a^
LD	0.99987	463.32 ± 28.34 ^ab^	461.20 ± 28.34 ^ab^	4.25 ± 0.12 ^a^	0.04 ± 0.00 ^a^
MET	0.99989	439.51 ± 45.70 ^b^	438.00 ± 45.50 ^b^	4.06 ± 0.12 ^a^	0.05 ± 0.00 ^a^

Note: Values are presented as mean ± SD (n = 4); different superscript letters indicate statistically significant differences among groups (*p* < 0.05). Identical letters denote no significant difference.

**Table 4:** Changes in SCFAs in T2D rats.

SCFAs (μg/mL)	NC	T2D	HD	LD	MET
Acetic acid	279.5675 ± 1.449 ^a^	206.930 ± 5.404 ^d^	271.848 ± 2.589 ^a^	248.92 ± 4.748 ^b^	226.375 ± 5.515 ^c^
Propionic acid	93.180 ± 1.406 ^c^	85.070 ± 1.819 ^c^	201.695 ± 3.228 ^a^	103.085 ± 1.674 ^b^	66.133 ± 1.524 ^d^
Butyric acid	64.438 ± 1.718 ^a^	48.618 ± 1.406 ^b^	68.690 ± 0.610 ^a^	51.300 ± 1.827 ^b^	53.253 ± 2.985 ^b^

Note: Values are presented as mean ± SD (n = 4); different superscript letters indicate statistically significant differences among groups (*p* < 0.05). Identical letters denote no significant difference.

## Data Availability

The original contributions presented in the study are included in the article, further inquiries can be directed to the corresponding author.
